# Supplementary material for: Robotic Rectal Resection for Rectal Cancer in Elderly Patients: A Systematic Review and Meta-Analysis
Source: J Clin Med. 2023 Aug 16;12(16):5331. doi: 10.3390/jcm12165331 (PMC10456068; doi:10.3390/jcm12165331)
Supplement: Supplementary file 1 [file jcm-12-05331-s001.zip › jcm-2494095-supplementary.pdf]

Table 1. suppl. Search strategies.

Database	Research srategy	Studies matched
**PubMed**	("Robotic Surgical Procedures"[Mesh] OR (("Robotics"[Mesh] OR robot*[tiab] OR Davinci*[tiab] OR "Da-Vinci*"[tiab] OR Senhance*[tiab] OR "Revo-I*"[tiab] OR Versius*[tiab] OR Avatera*[tiab] OR Hinotori*[tiab]) AND (surgery[subheading] OR surg*[tiab] OR resect*[tiab] OR remov*[tiab] OR excis*[tiab]))) AND ("Colorectal Neoplasms"[Mesh] OR ((rectum[tiab] OR rectal[tiab] OR anus[tiab] OR anal[tiab] OR colorect*[tiab] OR colo-rect*[tiab] OR anorect*[tiab] OR ano-rect*[tiab]) AND (cancer*[tiab] OR tumor*[tiab] OR tumour*[tiab] OR neoplas*[tiab] OR malignan*[tiab] OR carcinoma*[tiab]))) AND ("Aged"[Mesh] OR "Aging"[Mesh] OR aged[tiab] OR aging[tiab] OR elder*[tiab] OR geriatr*[tiab] OR gerontol*[tiab] OR old[tiab] OR older[tiab])	817
**Embase**	('robot assisted surgery'/exp OR 'robotic surgical procedure'/exp OR 'robotic surgical device'/exp OR (('robotics'/exp OR robot*:ti,ab,kw OR Davinci*:ti,ab,kw OR 'Da-Vinci*':ti,ab,kw OR Senhance*:ti,ab,kw OR 'Revo-I*':ti,ab,kw OR Versius*:ti,ab,kw OR Avatera*:ti,ab,kw OR Hinotori*:ti,ab,kw) AND (surgery:lnk OR surg*:ti,ab,kw OR resect*:ti,ab,kw OR remov*:ti,ab,kw OR excis*:ti,ab,kw))) AND ('colorectal tumor'/exp OR 'anorectal tumor'/exp OR ((rectum:ti,ab,kw OR rectal:ti,ab,kw OR anus:ti,ab,kw OR anal:ti,ab,kw OR colorect*:ti,ab,kw OR colo-rect*:ti,ab,kw OR anorect*:ti,ab,kw OR ano-rect*:ti,ab,kw) AND (cancer*:ti,ab,kw OR tumor*:ti,ab,kw OR tumour*:ti,ab,kw OR neoplas*:ti,ab,kw OR malignan*:ti,ab,kw OR carcinoma*:ti,ab,kw))) AND ('aged'/exp OR 'aging'/exp OR aged:ti,ab,kw OR aging:ti,ab,kw OR elder*:ti,ab,kw OR geriatr*:ti,ab,kw OR gerontol*:ti,ab,kw OR old:ti,ab,kw OR older:ti,ab,kw)	1595
**Cochrane CENTRAL**	#1 MeSH descriptor: [Robotic Surgical Procedures] explode all trees<br>#2 MeSH descriptor: [Robotics] explode all trees<br>#3 (robot* OR Davinci* OR "Da-Vinci*" OR Senhance* OR "Revo-I*" OR Versius* OR Avatera* OR Hinotori*):ti,ab,kw<br>#4 #2 OR #3<br>#5 MeSH descriptor: [] explode all trees and with qualifier(s): [surgery - SU]<br>#6 (surg* OR resect* OR remov* OR excis*):ti,ab,kw<br>#7 #5 OR #6<br>#8 #4 AND #7<br>#9 #1 OR #8<br>#10 MeSH descriptor: [Colorectal Neoplasms] explode all trees<br>#11 (rectum OR rectal OR anus OR anal OR colorect* OR colo-rect* OR anorect* OR ano-rect*):ti,ab,kw<br>#12 (cancer* OR tumor* OR tumour* OR neoplas* OR malignan* OR carcinoma*):ti,ab,kw<br>#13 #11 AND #12<br>#14 #10 OR #13<br>#15 MeSH descriptor: [Aged] explode all trees<br>#16 MeSH descriptor: [Aged] explode all trees<br>#17 (aged OR aging OR elder* OR geriatr* OR gerontol* OR old OR older):ti,ab,kw<br>#18 #15 OR #16 OR #17<br>#19 #9 AND #14 AND #18	89

Study or Subgroup	Elderly Mean	Elderly SD	Elderly Total	Young Mean	Young SD	Young Total	Weight	Mean Difference IV, Random, 95% CI
Oldani 2017	4.57	6.25	8	2.33	3.67	7	7.0%	2.24 [−2.87, 7.35]
Ramallo-Solis 2019	2.372	1.65	43	2.387	1.62	108	54.8%	−0.02 [−0.60, 0.57]
Su 2021	1.79	2.65	30	3.42	5.99	126	38.2%	−1.63 [−3.04, −0.22]
**Total (95% CI)**			**81**			**241**	**100.0%**	**−0.47 [−1.92, 0.97]**

Heterogeneity: $Tau^2 = 0.90$; $Chi^2 = 5.20$, df = 2 (P = 0.07); $I^2 = 62\%$
Test for overall effect: Z = 0.64 (P = 0.52)

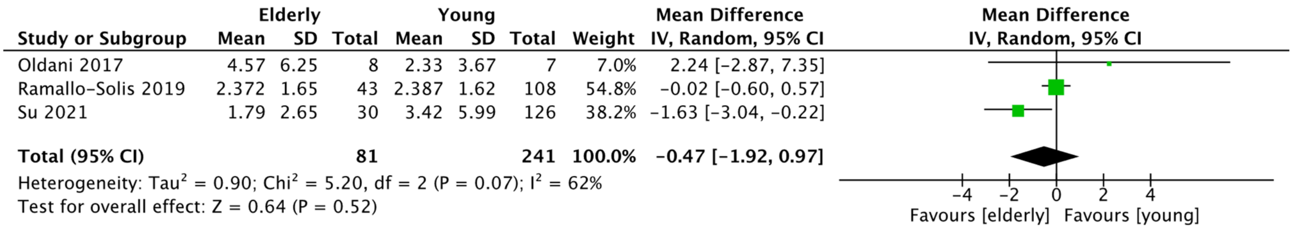

Figure 1. Distal Margin Forest Plot.

Study or Subgroup	Elderly Events	Elderly Total	Young Events	Young Total	Weight	Risk Difference M-H, Fixed, 95% CI
Oldani 2017	0	8	0	7	6.4%	0.00 [−0.22, 0.22]
Ramallo-Solis 2019	7	43	14	108	52.4%	0.03 [−0.09, 0.16]
Su 2021	0	30	0	126	41.3%	0.00 [−0.05, 0.05]
**Total (95% CI)**		**81**		**241**	**100.0%**	**0.02 [−0.05, 0.09]**
Total events	7		14			

Heterogeneity: $Chi^2 = 0.64$, df = 2 (P = 0.73); $I^2 = 0\%$
Test for overall effect: Z = 0.48 (P = 0.63)

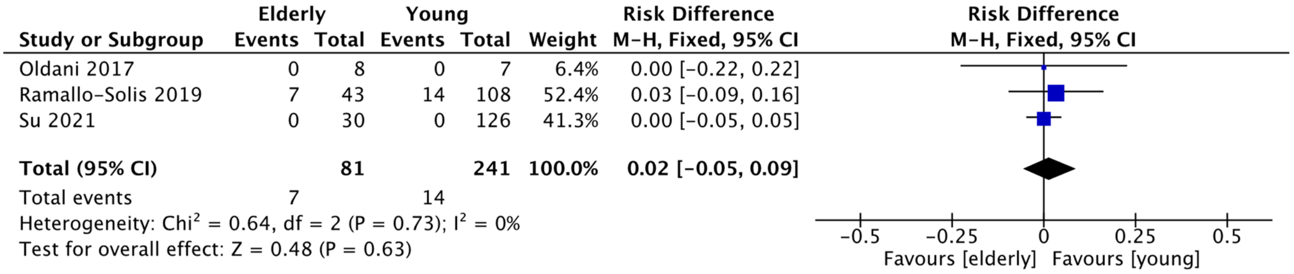

Figure 2. Conversion Rate Forest Plot.

Study or Subgroup	Elderly Events	Elderly Total	Young Events	Young Total	Weight	Odds Ratio M-H, Fixed, 95% CI
Oldani 2017	0	8	1	7	6.9%	0.25 [0.01, 7.34]
Ramallo-Solis 2019	12	43	29	108	54.8%	1.05 [0.48, 2.33]
Su 2021	5	30	26	126	38.3%	0.77 [0.27, 2.20]
**Total (95% CI)**		**81**		**241**	**100.0%**	**0.89 [0.48, 1.65]**
Total events	17		56			

Heterogeneity: $Chi^2 = 0.78$, df = 2 (P = 0.68); $I^2 = 0\%$
Test for overall effect: Z = 0.37 (P = 0.71)

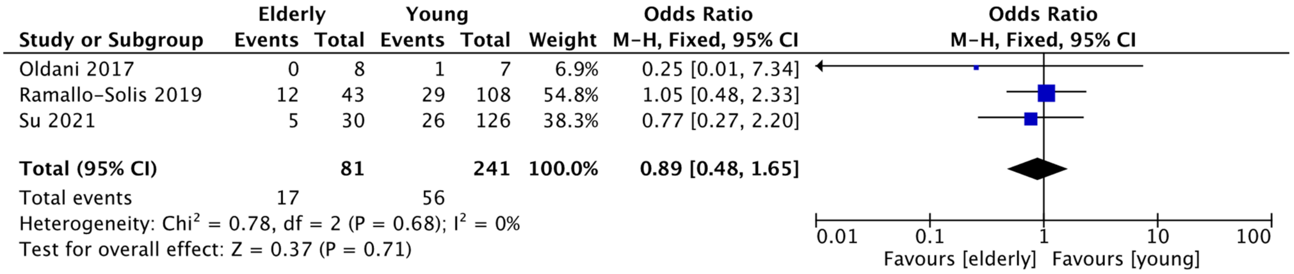

Figure 1. Complications Forest Plot.
